# The association between the systemic inflammation response index and overactive bladder: a cross-sectional study

**DOI:** 10.1186/s40001-025-02773-3

**Published:** 2025-06-16

**Authors:** Liandong Chen, Xier Xu, Yidong Zhou

**Affiliations:** 1 The Fifth School of Clinical Medicine, Zhejiang Chinese Medical University, Hangzhou, 310053 Zhejiang China; 2 The School of Pharmacy, Zhejiang Chinese Medical University, Hangzhou, 310053 Zhejiang China; 3 The Fourth School of Clinical Medicine, Zhejiang Chinese Medical University, 548 Binwen Road, Binjiang District, Hangzhou, 310053 Zhejiang China

**Keywords:** SIRI, OAB, NHANES, OABSS, Inflammation

## Abstract

**Background:**

Overactive bladder (OAB) is an intricate disorder with an unclear pathophysiological relationship with inflammation. This study employs the Systemic Inflammation Response Index (SIRI) as a quantitative measure of systemic inflammatory status and explores its association with both the risk and severity of OAB, as assessed by the Overactive Bladder Symptom Score (OABSS).

**Methods:**

Population data from the National Health and Nutrition Examination Survey (NHANES) in 2005–2020 were extracted. Weighted logistic regression and weighted linear regression models were utilized to examine the relation of SIRI to OAB risk and OABSS. The possible nonlinear relation of SIRI to clinical outcomes was examined via restricted cubic spline (RCS) models. In addition, subgroup analyses and interaction tests helped to explore the consistency of these associations across subpopulations.

**Results:**

23,915 individuals were encompassed for our analysis. 9011 (21%) were diagnosed with OAB. Both weighted linear and logistic regression analyses demonstrated a significant link of SIRI to OAB risk and symptom severity (fully adjusted model: *β* = 0.143, 95% CI 0.086–0.200, *P* < 0.001; OR = 1.268, 95% CI 1.122–1.413, *P* < 0.001). The RCS model showed a significant nonlinear relation of SIRI to clinical outcomes. Subgroup analyses further demonstrated the consistency of these associations across various subgroups.

**Conclusions:**

SIRI is a significant risk factor for OAB, and higher SIRI levels are strongly related to heightened OAB symptom severity.

## Introduction

Overactive bladder (OAB) is a urological syndrome primarily featuring urgency along with heightened urinary frequency and nocturia. This syndrome may or may not involve involuntary urine leakage and occurs without urinary tract infections or overt pathological changes [1]. Its pathogenesis is highly complex and involves multiple potential risk factors, including chronic anxiety and depression, neurological abnormalities, sex, obesity, aging, and hormonal fluctuations. In addition, lifestyle factors such as smoking, alcohol consumption, and high caffeine intake have been implicated in exacerbating OAB symptoms [2–4]. Urodynamically, OAB is often associated with detrusor overactivity, where involuntary bladder contractions occur before reaching normal bladder capacity, giving rise to frequent urgency [5]. Studies have demonstrated that OAB significantly impairs patients’ quality of life, resulting in physical discomfort, psychological distress, as well as social withdrawal. When compared with healthy individuals, OAB patients exhibit higher levels of anxiety and depressive symptoms, contributing to further deterioration of their overall health status [6]. Moreover, OAB imposes a substantial economic burden, encompassing direct medical costs like surgical interventions and pharmacotherapy, and indirect ones including productivity loss, caregiving expenses, and increased utilization of healthcare resources owing to frequent medical visits and long-term disease management [7]. Understanding the fundamental aspects of OAB and its influencing factors is crucial for developing targeted interventions at both individual and societal levels.

The Systemic Inflammation Response Index (SIRI), an emerging biomarker of systemic inflammation can more comprehensively reflect the inflammatory state by incorporating the ratio of neutrophils, monocytes, and lymphocytes in comparison to traditional single inflammatory markers [8]. SIRI has gained increasing recognition in clinical research, with elevated levels being related to the development and poor prognosis of pneumonia, cardiovascular diseases, cancer, and metabolic disorders, among other diseases [9–11]. For instance, in studies on pancreatic cancer and sepsis-associated acute kidney injury (SA-AKI), SIRI has demonstrated predictive potential for patient survival and tumor prognosis [12].

Mounting evidence reveals systemic inflammation’s crucial role in the pathogenesis of OAB [13]. Inflammatory mediators like cytokines and chemokines have been implicated in disrupting the urothelial barrier and impairing local neural signaling, with elevated levels potentially contributing to OAB symptoms [14, 15]. Given that SIRI reflects systemic inflammatory status, and its association with various diseases has been extensively investigated, its role and underlying mechanisms in OAB remain unclear. Whether SIRI can be a prospective biomarker and prognostic indicator for OAB has yet to be fully explored. Therefore, this study comprehensively examines the association of SIRI with OAB utilizing the National Health and Nutrition Examination Survey (NHANES) (2005–2020) data. By elucidating this relationship, our study aims to present novel insights into the pathogenesis, diagnosis, and prognostic evaluation of OAB.

## Method

### Data sources

NHANES is an ongoing, population-based, cross-sectional survey of the National Center for Health Statistics (NCHS) in America. Utilizing an intricate, multistage probability sampling design, NHANES collects nationally representative health and nutrition data from non-institutionalized Americans through structured interviews, physical examinations, and laboratory tests. These data encompass, but are not limited to, demographic characteristics (e.g., sex and age), physical activity, dietary intake, biomarkers, and the prevalence of chronic diseases. NHANES data are released biennially to the global research community, facilitating population-level investigations into the associations between potential risk factors and disease outcomes.

In this study, data spanning seven survey cycles from 2005 to 2020 were encompassed. Based on the data availability, our study excluded individuals with missing complete data on neutrophil count (NC), monocyte count (MC), or lymphocyte count (LC), as well as those lacking renal status information.

The NHANES program has been approved by the NCHS Ethics Review Board. Furthermore, patient information in this database has been anonymized, thereby obviating the need for informed consent.

### Assessment of SIRI

SIRI was derived based on the laboratory data provided by NHANES using the formula: SIRI = NC * MC/LC, where NC, MC, and LC were expressed in units of (1000 cells/µL) [16].

### Assessment of OAB

**Fig. 1:**
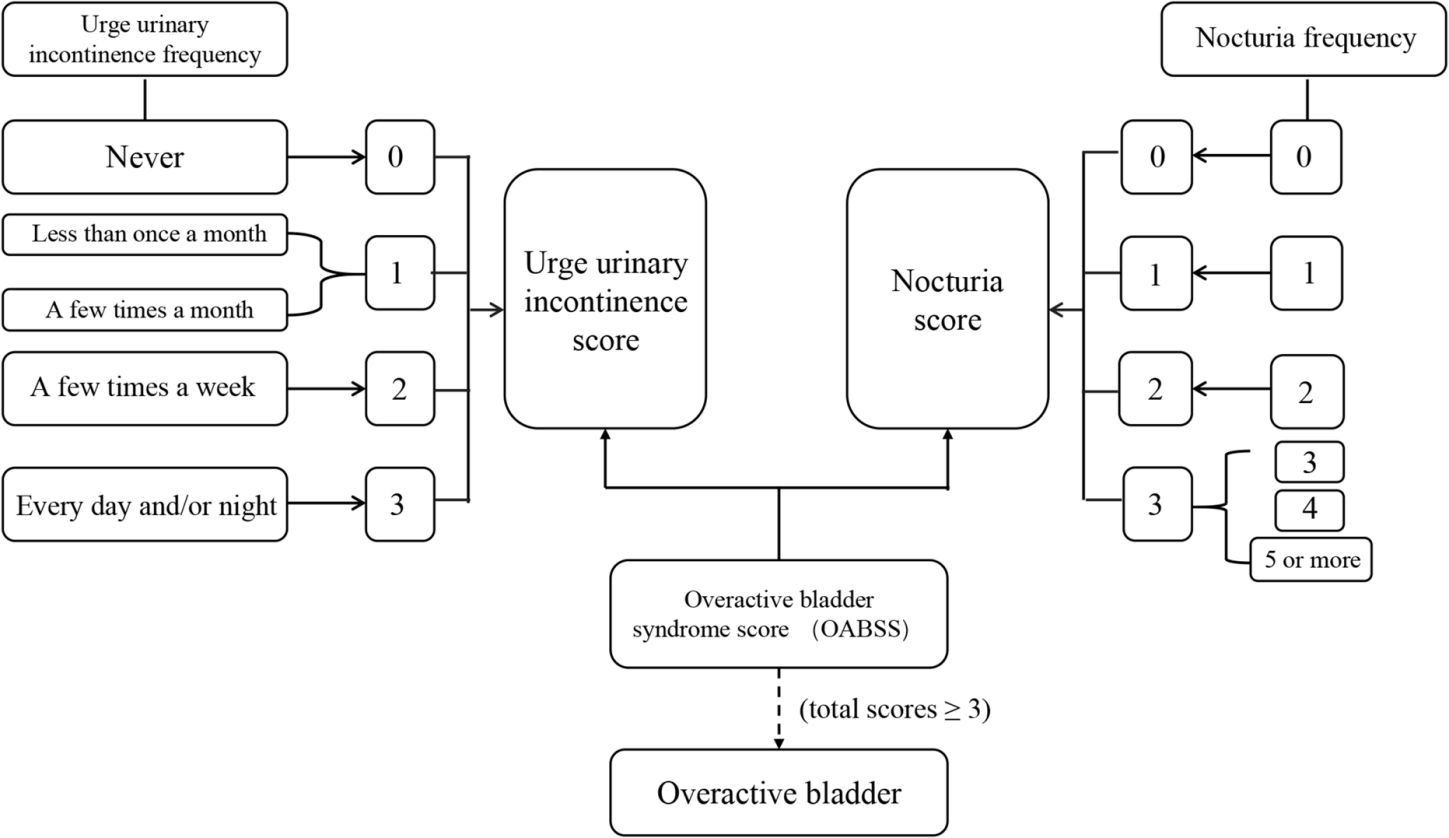
The process of OABSS

As defined by the International Continence Society (ICS), OAB is primarily characterized by nocturia and urge urinary incontinence (UUI). Therefore, the determination of OAB was based on the nocturia and UUI assessment [17]. The data on these symptoms were collected via structured questionnaires administered by trained research professionals. Participants were asked the following two questions: (1) “How many times do you urinate at night?”; (2) “How frequently do you experience urinary leakage?”. Based on the responses, the Overactive Bladder Symptom Score (OABSS) was employed for ascertaining OAB status [18]. Individuals with a combined nocturia and UUI score of ≥ 3 indicated OAB [19, 20], Fig. 1 summarizes the scoring criteria.

### Potential covariates

To control for potential confounders, our study incorporated covariates that may influence the link of SIRI to OAB. These were: demographic variables: sex (male/female) and age (< 40/40–60/> 60); laboratory variables: urinary albumin (U-Alb), creatinine, red blood cell (RBC), hemoglobin, glycated hemoglobin, total cholesterol(TC), triglycerides, and low-density lipoprotein cholesterol (LDL-C); behavioral and lifestyle factors: body mass index (BMI), pregnancy (yes/no), insulin injection use (yes/no), depression (yes/no), fatigue (yes/no), regular weekly work hours > 35 (yes/no), and self-reported poor health status (yes/no); clinical factors: hypertension (yes/no), hypercholesterolemia (yes/no), diabetes (yes/no), and renal failure(RF) (yes/no). Behavioral and clinical variables were collected through standardized questionnaires administered by professionally trained researchers. Demographic and laboratory data were obtained by healthcare technicians following standardized measurement protocols, which can be accessed at https://www.cdc.gov/nchs/nhanes.

For variables with less than 25% missing values, multiple imputations through a random forest-based approach were applied. Those with more than 25% missing values were categorized and incorporated into the model as dummy variables.

### Statistical analysis

For continuous variables in a normal distribution, the mean and standard deviation (SD) are reported, and between-group comparisons were performed utilizing the *t* test. For non-normally distributed ones, the median and interquartile range (IQR) are reported, and groups were compared via the Mann–Whitney U test. Categorical variables are shown in frequencies and percentages, with between-group differences assessed through the chi-square or Fisher’s exact test.

To unravel the relation of SIRI to OAB, weighted linear and logistic regressions were executed with OABSS and OAB as outcome variables. Three models were developed: Model 1 had no adjustments. Model 2 accounted for age and sex, while Model 3 additionally incorporated adjustments for BMI, pregnancy, RF, diabetes, and additional potential confounders not included in Model 2. For the multivariable models, the variance inflation factor (VIF) was computed for every variable and those with VIF > 5 to mitigate multicollinearity were excluded. In addition, restricted cubic spline (RCS) models were constructed to explore possible nonlinear relationships of exposure with outcomes.

To evaluate the robustness of the results, subgroup analyses and interaction tests were executed based on sex, pregnancy, and RF. All of our analyses were conducted using the survey package in R, accounting for the complex sampling design. In accordance with the NHANES database guidelines, the MEC weights were applied for weighting.

Every statistical analysis was enabled by R 4.4.2. A two-tailed *P* < 0.05 suggested statistical significance.

## Results

### Cohort study

**Fig. 2:**
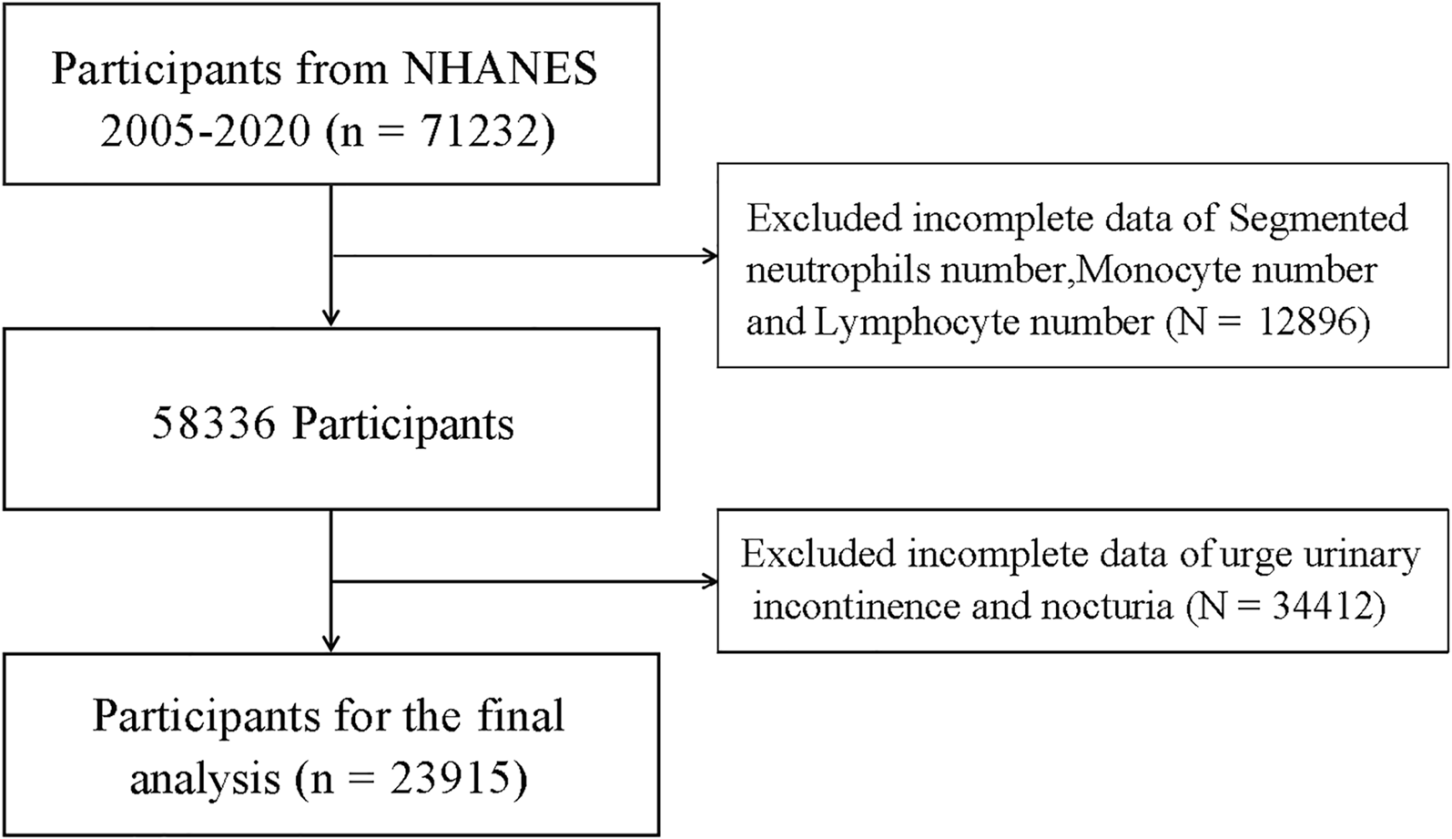
Patient selection flowchart

Owing to incomplete SIRI data, including missing values for LC, NC, and MC, 12,896 individuals were excluded. In addition, 34,912 individuals were excluded due to missing data on urgency urinary incontinence (UUI) and nocturia. Ultimately, 23,915 participants were included in the final analysis. The detailed cohort selection process is presented in Fig. 2.

Of all participants, women accounted for 51%, and most were aged 40–60. Individuals with OAB comprised 21% of the total population. In contrast to the lowest SIRI quartile (Q1), the highest quartile (Q4) had markedly higher prevalence rates of hypertension, hypercholesterolemia, diabetes, and OAB. In addition, laboratory parameters, including urine protein, creatinine, LC, MC, NC, and total blood cell count, were notably risen in the Q4 group (*P* < 0.05 for all) (Table 1).

**Table 1:** All characteristic of the participants (grouped by SIRI level)

Characteristic	Overall	Q1	Q2	Q3	Q4	*P* value^2^
	*N* = 223,799,286^1^	*N* = 31,041,685^1^	*N* = 55,705,767^1^	*N* = 66,885,269^1^	*N* = 70,166,565^1^	
Urine albumin	7 (4,15)	7 (4,13)	7 (4,13)	7 (4,14)	9 (5,19)	< 0.001***
Creatinine	9636 (5,392,14,674)	9812 (5,392,15,205)	9370 (5,039,14,498)	9547 (5,392,14,498)	9724 (5,569,14,851)	< 0.001***
BMI	28 (24,33)	27 (23,31)	28 (24,32)	28 (25,33)	29 (25,34)	< 0.001***
High blood pressure	13,165 (33%)	1888 (28%)	2890 (27%)	3621 (31%)	4766 (40%)	< 0.001***
High cholesterol level	12,965 (35%)	1925 (31%)	3071 (32%)	3715 (36%)	4254 (38%)	< 0.001***
Lymphocyte number	2.00 (1.60,2.50)	2.20 (1.80,2.70)	2.10 (1.70,2.60)	2.10 (1.70,2.50)	1.90 (1.50,2.30)	< 0.001***
Monocyte number	0.50 (0.40,0.70)	0.40 (0.30,0.50)	0.50 (0.40,0.50)	0.50 (0.50,0.60)	0.70 (0.60,0.80)	< 0.001***
Segmented neutrophils number	4.00 (3.10,5.10)	2.50 (2.00,3.00)	3.40 (2.90,3.90)	4.10 (3.50,4.90)	5.40 (4.60,6.60)	< 0.001***
Red blood cell count	4.69 (4.37,5.03)	4.61 (4.29,4.93)	4.67 (4.36,4.99)	4.71 (4.40,5.05)	4.72 (4.39,5.06)	< 0.001***
Hemoglobin	14.30 (13.30,15.30)	13.90 (13.00,14.90)	14.20 (13.30,15.10)	14.30 (13.40,15.40)	14.40 (13.30,15.50)	< 0.001***
Gender						< 0.001***
Female	18,284 (51%)	3421 (59%)	4828 (54%)	5125 (51%)	4910 (45%)	
Male	17,642 (49%)	2409 (41%)	4069 (46%)	5054 (49%)	6110 (55%)	
Age						< 0.001***
< 40 years	11,811 (36%)	2128 (42%)	3099 (39%)	3333 (35%)	3251 (31%)	
> 60 years	11,510 (25%)	1454 (19%)	2424 (20%)	3162 (25%)	4470 (34%)	
0–60 years	12,605 (39%)	2248 (40%)	3374 (41%)	3684 (40%)	3299 (36%)	
Pregnant						< 0.001***
Missing	27,985 (77%)	4385 (73%)	6822 (74%)	7927 (77%)	8851 (80%)	
No	7337 (22%)	1419 (27%)	2034 (25%)	2156 (23%)	1728 (18%)	
Yes	604 (1.1%)	26 (0.3%)	41 (0.4%)	96 (0.7%)	441 (2.4%)	
Diabetes						< 0.001***
Borderline	850 (2.1%)	146 (2.4%)	184 (1.6%)	235 (1.9%)	285 (2.6%)	
No	30,298 (88%)	5064 (90%)	7692 (91%)	8631 (89%)	8911 (84%)	
Yes	4778 (10.0%)	620 (7.3%)	1021 (7.7%)	1313 (9.4%)	1824 (13%)	
Taking insulin	1359 (2.8%)	134 (1.6%)	257 (1.8%)	363 (2.5%)	605 (4.3%)	< 0.001***
Depressed						0.053
More than half the days	1480 (3.5%)	255 (3.7%)	331 (3.1%)	413 (3.5%)	481 (3.7%)	
Nearly every day	1225 (2.6%)	172 (2.4%)	285 (2.5%)	325 (2.4%)	443 (3.1%)	
Not at all	27,139 (77%)	4406 (77%)	6800 (78%)	7741 (78%)	8192 (76%)	
Several days	6082 (17%)	997 (17%)	1481 (16%)	1700 (16%)	1904 (17%)	
Tired						< 0.001***
More than half the days	2903 (8.1%)	450 (7.3%)	678 (8.0%)	818 (8.2%)	957 (8.5%)	
Nearly every day	3094 (7.7%)	422 (6.6%)	649 (6.3%)	869 (7.9%)	1154 (9.2%)	
Not at all	18,160 (49%)	3066 (51%)	4582 (50%)	5188 (49%)	5324 (48%)	
Several days	11,769 (35%)	1892 (35%)	2988 (36%)	3304 (35%)	3585 (34%)	
Feeling bad about yourself						0.5
More than half the days	1014 (2.5%)	175 (2.5%)	242 (2.5%)	279 (2.3%)	318 (2.7%)	
Nearly every day	973 (2.2%)	149 (2.2%)	210 (2.1%)	274 (2.1%)	340 (2.5%)	
Not at all	29,869 (84%)	4845 (84%)	7410 (83%)	8534 (84%)	9080 (83%)	
Several days	4070 (12%)	661 (11%)	1035 (12%)	1092 (11%)	1282 (12%)	
Glycohemoglobin	5.40 (5.20,5.80)	5.40 (5.10,5.70)	5.40 (5.20,5.70)	5.40 (5.20,5.80)	5.50 (5.20,5.80)	< 0.001***
Weak kidneys	1211 (2.7%)	130 (2.1%)	216 (1.8%)	306 (2.4%)	559 (3.9%)	< 0.001***
Nocturia frequency						< 0.001***
0	11,004 (34%)	1842 (34%)	2918 (36%)	3259 (35%)	2985 (30%)	
1	13,200 (39%)	2181 (40%)	3405 (40%)	3791 (40%)	3823 (37%)	
2	6720 (16%)	1062 (15%)	1545 (14%)	1856 (16%)	2257 (19%)	
3	3194 (7.0%)	472 (6.6%)	657 (5.8%)	823 (5.8%)	1242 (9.2%)	
4	1028 (2.0%)	157 (1.8%)	222 (1.6%)	254 (1.8%)	395 (2.5%)	
5	780 (1.6%)	116 (1.5%)	150 (1.4%)	196 (1.5%0)	318 (2.0%)	
Usually work 35 or more hours per week						0.056
Missing	30,505 (84%)	4858 (82%)	7531 (84%)	8627 (84%)	9489 (84%)	
No	3371 (10%)	576 (11%)	870 (10%)	966 (9.7%)	959 (9.7%)	
Yes	2050 (6.3%)	396 (7.2%)	496 (6.0%)	586 (6.4%)	572 (5.9%)	
Total cholesterol	4.91 (4.24,5.64)	4.91 (4.27,5.66)	4.97 (4.29,5.66)	4.94 (4.27,5.64)	4.81 (4.14,5.56)	< 0.001***
Triglyceride						< 0.001***
< 0.45	691 (2.1%)	201 (4.2%)	197 (2.5%)	157 (1.7%)	136 (1.3%)	
> 1.69	4327 (12%)	607 (10%)	1173 (13%)	1179 (12%)	1368 (13%)	
0.45–1.69	12,058 (35%)	2383 (43%)	3154 (38%)	3280 (35%)	3241 (31%)	
Missing	18,850 (50%)	2639 (42%)	4373 (47%)	5563 (51%)	6275 (55%)	
LDL cholesterol						< 0.001***
< 2.07	3080 (8.6%)	546 (9.9%)	673 (7.7%)	787 (7.7%)	1074 (9.7%)	
> 3.37	4820 (14%)	978 (18%)	1355 (15%)	1312 (15%)	1175 (11%)	
2.07–3.37	8901 (26%)	1624 (29%)	2405 (29%)	2447 (25%)	2425 (24%)	
Missing	19,125 (51%)	2682 (43%)	4464 (48%)	5633 (52%)	6346 (56%)	
SIRI	1.05 (0.73,1.54)	0.46 (0.36,0.52)	0.75 (0.67,0.82)	1.11 (1.00,1.23)	1.89 (1.58,2.41)	< 0.001***
OABSS						< 0.001***
0	8815 (27%)	1515 (28%)	2327 (29%)	2614 (28%)	2359 (24%)	
1	10,976 (33%)	1837 (34%)	2899 (34%)	3164 (34%)	3076 (30%)	
2	7124 (19%)	1146 (19%)	1691 (18%	2063 (19%)	2224 (20%)	
3	5060 (12%)	798 (12%)	1164 (11%)	1281 (10%)	1817 (14%)	
4	2158 (5.3%)	329 (5.2%)	460 (4.6%)	571 (5.0%)	798 (6.1%)	
5	1020 (2.4%)	126 (1.7%)	205 (1.9%)	278 (2.2%)	411 (3.2%)	
6	773 (1.6%)	79 (1.1%)	151 (1.2%)	208 (1.4%)	335 (2.5%)	
OAB	9011 (21%)	1332 (20%)	1980 (19%)	2338 (19%)	3361 (26%)	< 0.001***

*OAB* Overactive bladder, *BMI* Body mass index, *LDL* Low density lipoprotein, *SIRI* systemic inflammation response index

^1^ Median (Q1, Q3); *n* (unweighted) (%)

^2^ *P < 0.05; ** *P* < 0.01; *** *P* < 0.001

### Association of SIRI with OAB and OABSS

The relationship between SIRI, OAB, and OABSS was analyzed through logistic regression. Initially, SIRI was treated as a continuous variable in the model. As presented in Table 2, the odds ratios (ORs) for SIRI in Models 1, 2, and 3 were OR = 1.238 (95% CI 1.201–1.276), OR = 1.215 (95% CI 1.172–1.260), and OR = 1.171 (95% CI 1.130–1.213). These findings indicate a positive link of higher SIRI levels to an increased likelihood of OAB among the unadjusted, partially adjusted, and fully adjusted models. Subsequently, SIRI was categorized into quartiles (IQR) and included in the model. When compared with individuals in Q1, those in Q4 exhibited a notably higher likelihood of OAB. The ORs for the unadjusted, partially adjusted, and fully adjusted models were OR = 1.453 (95% CI 1.304–1.619), OR = 1.371 (95% CI 1.219–1.542), and OR = 1.268 (95% CI 1.122–1.434).

**Table 2:** Association of SIRI with OAB and OABSS

Characteristic	Model 1			Model 2			Model 3		
	Beta/OR	95% CI	*P* value	Beta/OR	95% CI	*P* value	Beta/OR^1^	95% CI	*P* value
OAB									
SIRI	1.238	1.201, 1.276	< 0.001	1.215	1.172, 1.260	< 0.001	1.171	1.130, 1.213	< 0.001
SIRI(IQR)	p of trend: < 0.001			p of trend: < 0.001			p of trend: 0.001		
Q1	–	–		–	–		–	–	
Q2	0.955	0.847, 1.076	0.445	0.96	0.846, 1.088	0.518	0.968	0.853, 1.098	0.613
Q3	0.969	0.861, 1.089	0.591	0.929	0.822, 1.050	0.234	0.908	0.800, 1.030	0.132
Q4	1.453	1.304, 1.619	< 0.001	1.371	1.219, 1.542	< 0.001	1.268	1.122, 1.434	< 0.001
OABSS									
SIRI	0.152	0.134, 0.170	< 0.001	0.115	0.098, 0.132	< 0.001	0.079	0.063, 0.094	< 0.001
SIRI(IQR)	p of trend: < 0.001			p of trend: < 0.001			p of trend: < 0.001		
Q1	–	–	–	–	–		–	–	
Q2	− 0.028	− 0.092, 0.036	0.386	− 0.023	− 0.083, 0.037	0.444	0.003	− 0.054, 0.060	0.918
Q3	0.019	− 0.050, 0.088	0.586	− 0.01	− 0.073, 0.052	0.74	0.006	− 0.056, 0.068	0.847
Q4	0.268	0.203, 0.332	< 0.001	0.188	0.130, 0.246	< 0.001	0.143	0.086, 0.200	< 0.001

*CI* confidence interval, *OR* odds ratio, *OAB* overactive bladder

Model 1 was unadjusted. Model 2 was adjusted for age and gender. Model 3 was adjusted for age, gender, diabetes, pregnant, taking insulin, depressed, weak kidneys, usually working 35 or more hours per week, triglyceride, LDL cholesterol, and high blood pressure

Q1: 0–0.577, Q2: 0.577–0.900, Q3: 0.900–1.375, Q4: 1.375–29.043

Weighted linear regression analysis demonstrated that when SIRI served as a continuous variable, higher SIRI levels notably correlated with greater OABSS scores: unadjusted model [*β* = 0.152 (95% CI 0.134–0.170)], partially adjusted model [*β* = 0.115 (95% CI 0.098–0.132)], and fully adjusted model [*β* = 0.079 (95% CI 0.063–0.094)]. When SIRI was analyzed as a categorical variable, individuals in Q4 had evidently higher OABSS scores than those in Q1: unadjusted model [*β* = 0.268 (95% CI 0.203–0.332)], partially adjusted model [*β* = 0.188 (95% CI 0.130–0.246)], and fully adjusted model [*β* = 0.143 (95% CI 0.086–0.200)] (Table 2).

### Potential nonlinear relationship between SIRI and OAB

**Fig. 3:**
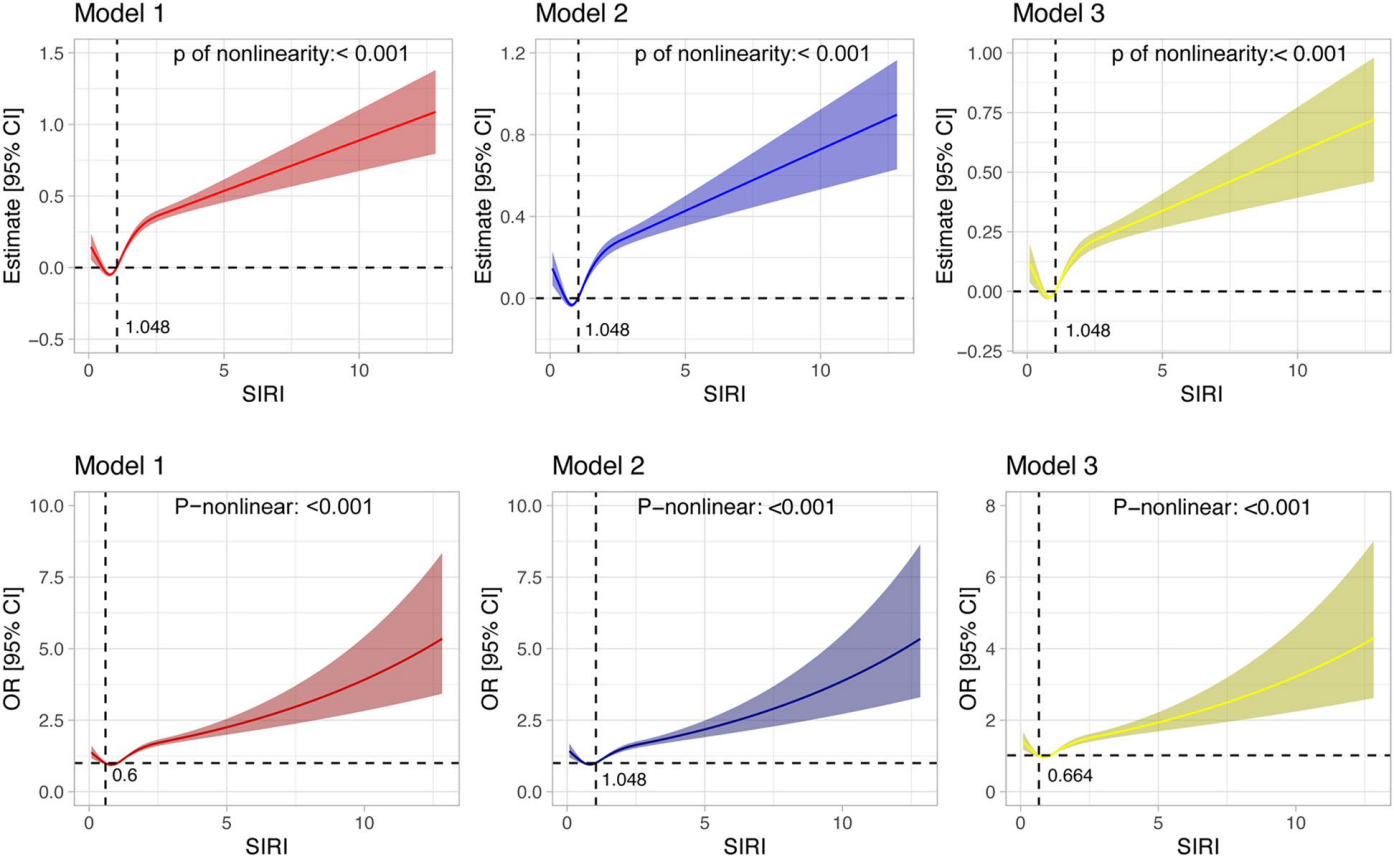
Results of RCP analysis. Model 1 was unadjusted. Model 2 was adjusted for age and sex, Model 3 was adjusted for Diabetes, hemoglobin, Pregnant, Taking insulin, Depressed, Weak kidneys, Glycohemoglobin, Usually working 35 or more hours per week, Total cholesterol, triglyceride, LDL cholesterol, High blood pressure

RCS analysis was conducted to examine the potential nonlinear relationship between SIRI and outcome measures. As shown in Fig. 3, SIRI exhibited a notable nonlinear association with both OABSS and OAB risk (*P* < 0.001). In the weighted linear regression analysis, when the Beta value was zero, the corresponding SIRI value was 1.048. This indicates that in all models, OABSS showed an approximately linear increase with rising SIRI levels once SIRI exceeded 1.048. Similarly, in the weighted logistic regression analysis, when the OR value equaled 1, the SIRI threshold in the unadjusted model was 0.6, beyond which the OAB risk increased in an almost linear manner as SIRI levels rose. In the fully adjusted model, the threshold corresponding to an OR of 1 was SIRI = 0.664, beyond which the risk of OAB also exhibited an approximately linear increase with rising SIRI levels.

### Subgroup analysis

**Fig. 4:**
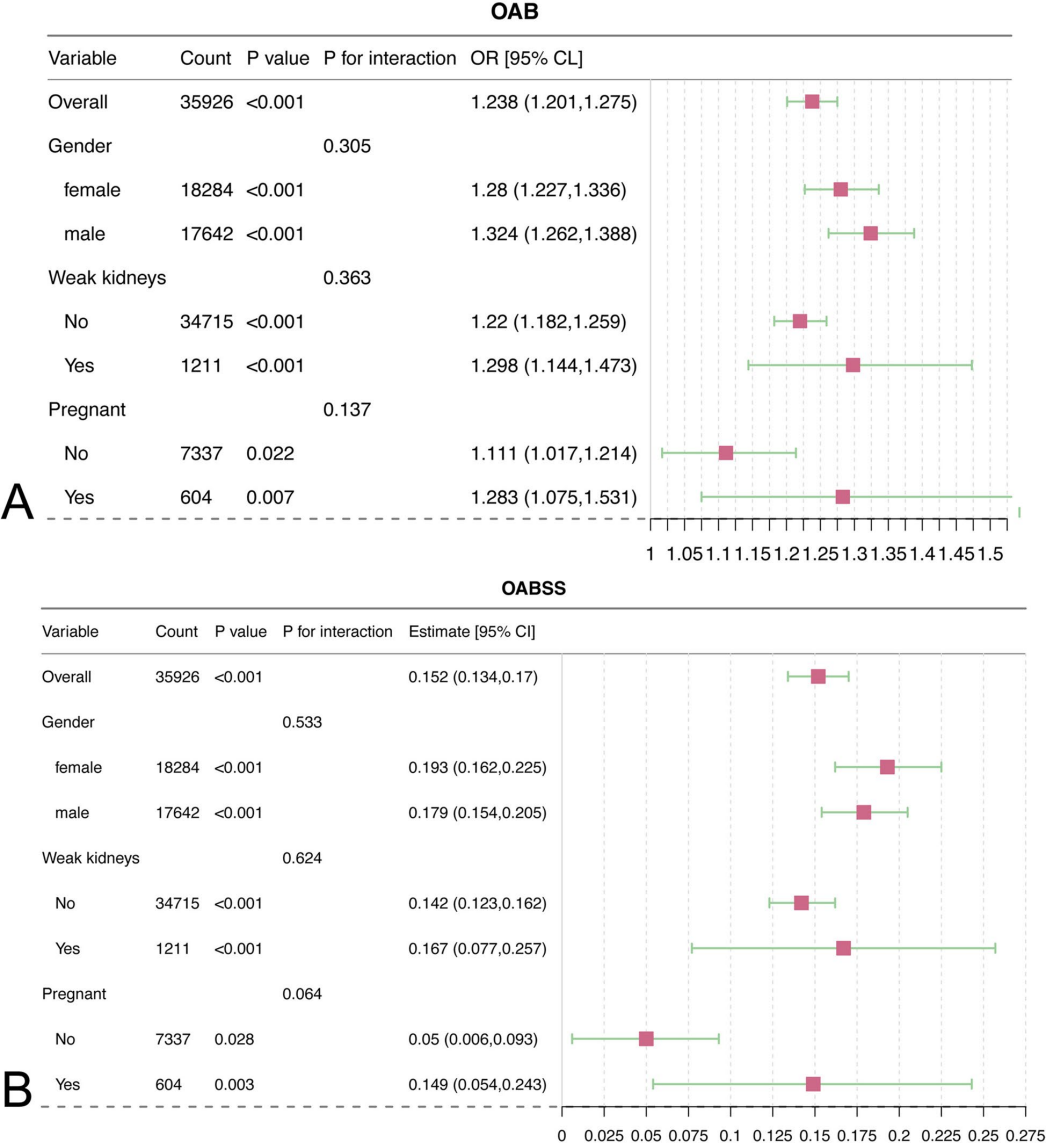
Results from subgroup analysis. **A** The outcome was the OAB. **B** The outcome was the OABSS. *OAB* overactive bladder, *OABSS* overactive bladder syndrome score

For variables exhibiting statistical significance in the weighted logistic regression analysis (sex, RF, and pregnancy), subgroup analyses were carried out. Figure 4 indicated that the connection of SIRI with both OAB and OABSS remained significant across all subgroups, exhibiting a high degree of consistency regardless of sex, the presence of RF, or pregnancy in female patients. Furthermore, significant interaction was not noted between SIRI and these subgroups.

## Discussion

This study is a cross-sectional investigation based on the NHANES database, aimed at exploring the relationship of SIRI with OAB. Our findings indicate a significant positive correlation between SIRI levels and both the risk and severity of OAB in the study population, with this association remaining robust even after adjusting for potential confounders. OAB is a common condition affecting both men and women, characterized by complex symptoms and pathophysiology, and it significantly impacts patients’ quality of life [21]. Given the sex-specific differences and additional factors influencing OAB, the development of effective treatment strategies and research into its pathogenesis have been challenging [22]. Although personalized medications for OAB are being developed, these therapies still lack universal applicability and are often accompanied by considerable side effects [23]. Therefore, a crucial focus of current research is to investigate the pathogenesis of OAB or identify risk factors contributing to its development, thereby optimizing its evaluation and improving clinical diagnostic and intervention strategies.

Inflammation, as a fundamental process underlying various physiological and pathological activities in the human body, is influenced by factors such as infection and tissue damage, which lead to the recruitment and residence of cytokines, chemokines, leukocytes, macrophages, and plasma proteins, as well as functional disorders and stress responses. These processes may be closely associated with the pathogenesis of many urological diseases [24–27]. Plenty of studies have proved the link of inflammation to OAB via various pathways, including patients with chronic urinary inflammatory diseases experience altered sensitivity of the central nervous system’s perception of urination due to stress, anxiety, depression, and urinary dysfunction [28]. In OAB and chronic pelvic pain syndrome/prostatitis, cytokines and inflammatory cell recruitment induced by inflammation trigger pain symptoms and are considered one of the possible pathogenic factors [29]. Urodynamic studies have found that abnormal detrusor activity, bladder outlet obstruction, mixed urinary incontinence, as well as higher urinary nerve growth factor (NGF) levels in the OAB population highly correlated with the severity of the disease [30]. Bacterial infections and urinary dysfunction further compromise the urothelial barrier in immunocompromised patients, ultimately leading to the activation of sensory nerves due to defects in the urothelial barrier function and basal proliferation caused by urinary inflammation, further triggering OAB [31].

SIRI, derived from LC, MC, and NC, serves as a quantitative indicator of systemic inflammation, offering new insights into disease mechanisms. Many studies have revealed a potential connection of SIRI with the risk and prognosis of conditions such as pneumonia, stroke, cancer, chronic kidney disease, type 2 diabetes, peripheral vascular disease, and sepsis [32–35]. This study utilized SIRI as an inflammatory marker to examine its correlation with OAB incidence and OABSS scores. Our analysis proves a notably positive relation of SIRI to both OAB incidence and severity, with consistent trends observed across various population subgroups. In addition, RCS analysis indicates a near-linear positive correlation between SIRI and both OAB and OABSS in both partially adjusted and fully adjusted models.

In conclusion, this study first elucidated a significant relation of SIRI to both the risk and severity of OAB. As a novel biological marker of inflammation, SIRI can assist clinicians in the early identification of patients at high risk for OAB, enabling timely intervention and treatment. This can optimize the utilization and rational distribution of healthcare resources, alleviating the burden on clinical practice. Moreover, as a refined indicator directly reflecting inflammation, SIRI may possess certain advantages over other traditional biomarkers or inflammatory indices. For instance, C-reactive protein (CRP), a classic inflammatory marker, has been studied for its predictive role in OAB but can only indirectly reflect the degree of chronic inflammation associated with OAB. Other studies have shown that few biomarkers independently assist in the diagnosis of OAB; rather, the combination of biomarkers such as CRP, nerve growth factor (NGF), brain-derived neurotrophic factor (BDNF), and prostaglandins are required to achieve greater specificity to accommodate the complexity of OAB [36, 37]. Similarly, studies exploring the association between the neutrophil-to-lymphocyte ratio (NLR) and OAB suggest that, due to the relatively simple calculation of NLR, it is susceptible to multiple confounding factors, including sex, age, hormone levels, and ethnicity, thereby introducing greater uncertainty in the identification of OAB patients [38]. Furthermore, the measurement of SIRI can enhance awareness of other inflammation-related diseases, promoting a holistic approach to health and providing a foundation for future in-depth studies.

Nevertheless, our limitations should be noted. First, since this is a cross-sectional study, a causal relationship of SIRI with OAB risk cannot be established. Furthermore, since the NHANES data were collected at a single point in time, it is subject to various fluctuating factors, and the subsequent changes and persistence of inflammation levels over time could not be further tracked or assessed. Prospective cohort studies are necessitated for further exploration, and longitudinal studies should be prioritized in the future investigations to unveil these unclear relationships. Furthermore, due to the reliance on self-reported data from the NHANES questionnaire, there may be recall bias and potential misclassification in the measurement of OABSS and confounding factors. Second, our study is based on Americans, and the findings may not apply to regions with significant differences in geography or lifestyle habits. In addition, due to the limited types of variables available in the NHANES database, certain factors of interest, such as genetics, imaging, and other bladder diseases, were not included in the analysis. Although various covariates have been adjusted for and incorporated, factors such as caffeine intake and prolonged sedentary behavior remain unexplained, which could significantly alter the results. The presence of residual confounding factors must be acknowledged, as it may influence future related sensitivity analyses. An unavoidable limitation is that, due to the missing data for essential variables such as OAB and SIRI, a portion of the study population was excluded, which may have introduced some degree of selection bias. Finally, the study does not unveil the mechanisms linking SIRI to OAB symptoms. Moreover, as OAB is a symptom complex rather than a singular disease entity, its influencing factors are not limited to systemic inflammation. Therefore, investigating the association between the SIRI and OAB leveraging the NHANES database may fail to fully capture the underlying pathophysiological mechanisms. As an inflammatory marker, SIRI represents a common pathway shared by various pathological processes, such as bladder outlet obstruction and peripheral neuropathy.

## Conclusion

An elevated SIRI is significantly linked to an elevated risk of developing OAB, with consistent trends observed across various subgroups. These findings could help clinicians identify high-risk populations for OAB at an early stage. However, further validation in prospective cohorts is necessitated.

## Data Availability

All data generated or analyzed during the course of this study were obtained from the NHANES database.
